# Effects of Wall Material on Medium-Chain Triglyceride (MCT) Oil Microcapsules Prepared by Spray Drying

**DOI:** 10.3390/pharmaceutics14061281

**Published:** 2022-06-16

**Authors:** Su Mon San, Montree Jaturanpinyo, Waree Limwikrant

**Affiliations:** Department of Manufacturing Pharmacy, Faculty of Pharmacy, Mahidol University, Bangkok 10400, Thailand; autumsunsetbreeze@gmail.com (S.M.S.); montree.jat@mahidol.ac.th (M.J.)

**Keywords:** medium-chain triglyceride oil, microencapsulation, microcapsule, spray drying, wall material, encapsulation efficiency

## Abstract

A medium-chain triglyceride (MCT) oil microcapsule was prepared by spray drying. The effects of the wall-material parameters of wall-to-oil ratio (1:1 to 3:1) and type of wall material (gum arabic (GA), whey protein isolate (WPI), and octenyl succinic anhydride (OSA) starch) on the microcapsules were evaluated. The droplet size, size distribution, viscosity, zeta potential, and stability of the emulsions were measured. The spray-dried powder was characterized by its morphology, yield, encapsulation efficiency, and moisture content. The wall material influenced the characteristics of the emulsions and microcapsules. The formulation with a 2:1 wall-to-oil ratio and OSA starch/maltodextrin as the wall material resulted in a small droplet size (0.177 ± 0.002 µm) with high encapsulation efficiency (98.38 ± 0.01%). This formulation had good physical stability over three months under accelerated conditions. Thus, OSA starch/maltodextrin is an appropriate wall material for encapsulating MCT oil.

## 1. Introduction

The medium-chain triglyceride (MCT) oil is a type of dietary triglyceride that is synthetically produced from rich resources, such as palm kernel oil and coconut oil, by fractionation [1,2]. The predominant components are caprylic (C8) and capric (C10) fatty acids (53% and 36–47%, respectively) with lesser amounts of caproic (C6) and lauric (C12) fatty acids [3]. MCT oil is a saturated oil that is flavorless with a low melting point (C8: 16.7 °C, C10: 31.3 °C). MCT oil can be metabolized more rapidly into ketone bodies and absorbed more efficiently than long-chain triglycerides [4]. In the 1950s, MCT oil was introduced as a clinical nutrient for the treatment of malabsorption syndromes [5]. The biggest hurdle in incorporating oils into various food products is their limited water solubility. The conversion of oil into powder is expected to provide a more convenient nutritional supplement.

In this regard, microencapsulation is a promising technique [6]. Microencapsulation with spray drying has been applied to food production on an industrial scale. Wall materials play a key role in microencapsulation, especially because they act as a barrier between external factors and core materials. Gum arabic (GA) is a highly branched edible polymer extracted from acacia trees. GA is comprised of approximately 2% protein and many carbohydrates, including d-glucuronic acid, d-galactose, l-arabinose, and L-rhamnose. GA is one of the most commonly used wall materials owing to its excellent emulsifying properties and ability to form stable emulsions over a wide pH range [7]. Whey protein isolate (WPI) is an attractive encapsulating agent for food products, especially fats, oils, and volatile compounds, owing to its amphiphilic properties [8]. Octenyl succinic anhydride (OSA) starch is modified by esterification with OSA to form a hydrocolloid with amphiphilic properties. This modified starch can be applied as an emulsifier and as a good thickening agent. Bearing free COO-charged groups in the side chain confers pronounced resistance to harsh environmental conditions to OSA starch via steric stabilization [9]. Maltodextrin (MD) is a widely used wall material. The distinct advantages of MD are its neutral taste and aroma, low viscosity at high solid content, low cost, and feasibility for rapid digestion [10]. However, the poor emulsifying property of MD decreases its tendency to be used as a wall material alone [11]. Therefore, MD is generally used in combination with other wall materials to overcome these shortfalls [12,13].

The influence of formulation factors on the droplet size distribution of MCT/zein microcapsules prepared by the antisolvent technique has been reported [14]. Minimal information is available on the microencapsulation of MCT oil, and none of the published works reported the influence of different types of wall materials on the encapsulation efficiency (EE) of microcapsules prepared by spray drying. Hence, the objectives of this study were to prepare MCT oil microcapsules by spray drying and to evaluate the effects of wall material (type of wall material and wall-to-oil ratio) on the physicochemical properties of emulsion and spray-dried microcapsules.

## 2. Materials and Methods

### 2.1. Materials 

MCT oil was obtained from Wilmar International Ltd. (Singapore). GA was purchased from Starlight Products (Rouen, France). MD was supplied by Banpong Novitat Co., Ltd. (Ratchaburi, Thailand). WPI was obtained from Davisco Foods International Inc. (Le Sueur, MIN, USA). OSA starch was obtained from Ingredion Inc. (Westchester, IL, USA). Anhydrous sodium sulfate crystals (Carlo-Erba, Cornaredo, Italy), hexane (Burdick and Jackson, Muskegon, MI, USA), and propan-2-ol (RCI Labscan, Bangkok, Thailand) were of analytical grade. 

### 2.2. Preparation of Emulsions 

A full factorial experimental design was used to study the effects of the wall-to-oil ratio (1:1, 2:1, and 3:1) and the type of wall material (GA, WPI, and OSA starch) on the characteristics of the emulsions and microcapsules (Table 1). MD was also used as a wall-material blend in all formulations at a fixed ratio of 1:1, and the total solid content was fixed at 40%. The wall materials were dissolved in deionized water using a magnetic stirrer and hydrated overnight at ambient temperature to ensure complete dissolution. The coarse emulsion was prepared by adding MCT oil to the wall solution, homogenizing with an Ultra-Turrax T25 high-speed homogenizer (IKA, Staufen, Germany) at 15,000 rpm for 3 min, followed by passage through a model APV-2000 high-pressure homogenizer (SPX Flow, Charlotte, NC, USA) at 500 bar for three cycles. The resulting emulsion was maintained at ambient temperature for 24 h and checked for stability.

### 2.3. Characterization of Emulsions

#### 2.3.1. Stability

The phase separation of the emulsions was checked by visual observation. The stability of the emulsions was determined using the following equation, where H_0_ is the initial height of the emulsion and H_1_ is the upper phase height.

% Creaming index = (H_1_/H_0_) × 100 (1)

#### 2.3.2. Droplet Size and Size Distribution 

Freshly prepared emulsions were characterized using a Mastersizer 2000 laser diffraction particle size analyzer (Malvern Panalytical Ltd., Malvern, UK). The refractive index of the sample and dispersant was 1.52 and 1.33, respectively. The droplet size was expressed as the mean volumetric size (D _[4,3]_; De Brouckere mean diameter) and was calculated using the following equation:

D _[4,3]_ = (Σnidi^4^)/(Σnidi^3^) (2)

where n_i_ is the number of particles of diameter d_i_, and by:

Span = (d_90_ − d_10_)/d_50_ (3)

where d_10_, d_50_, d_90_ are the equivalent volume diameters at 10%, 50%, 90% cumulative volumes, respectively. A smaller span value indicates a narrower size distribution. 

#### 2.3.3. Viscosity 

The viscosity of emulsions was measured using a Haake RotoVisco 1 rheometer (Thermo Fisher Scientific, Waltham, MA, USA) equipped with a Z34 DIN sensor (rotor: 17 ± 0.004 mm in radius and 51 ± 0.06 mm in length, and cup: 18.44 ± 0.004 mm in radius) operating at 500 rpm. The temperature was maintained at 30 °C using a thermostatic water bath. 

#### 2.3.4. Zeta Potential 

The zeta potentials of the emulsions were analyzed using a Zetasizer Nano-ZS photon correlation spectrophotometer (Malvern Panalytical Ltd., Malvern, UK). The samples were diluted 100 times with deionized water to avoid multiple scattering. All measurements were performed in triplicate.

### 2.4. Microencapsulation by Spray Drying

The emulsions were spray-dried using a model B-290 spray dryer (Büchi Labortechnik AG, Flawil, Switzerland) equipped with a standard nozzle (diameter: nozzle cap 1.5 mm, nozzle tip 0.7 mm, needle 0.7 mm). Feed flow rate and aspirator were set to 10% and 90%. Compressor air pressure for the spray flow was adjusted to 6 bar, while the inlet temperature was maintained at 150 °C. The outlet temperature was 95 ± 5 °C. The obtained spray-dried powder was stored at 2–8 °C until further analysis.

### 2.5. Characterization of Spray-Dried Powder

#### 2.5.1. Yield

Yield (%) was calculated as:

(4) $$\mathrm{Yield}=\frac{\mathrm{Weight}\;\mathrm{of}\;\mathrm{obtained}\;\mathrm{powder}\;\times \;100}{\mathrm{Total}\;\mathrm{weight}\;\mathrm{of}\;\mathrm{the}\;\mathrm{solid}\;\mathrm{content}\;\mathrm{in}\;\mathrm{the}\;\mathrm{initial}\;\mathrm{formulation}}$$

#### 2.5.2. Moisture Content 

Powder (0.5 g) was evenly spread over the aluminum pan of the model MA45 moisture analyzer (Sartorius AG, Göttingen, Germany) and heated at 105 °C. The drying was automatically stopped when a constant weight was obtained. Moisture content was presented in percentage (%). 

#### 2.5.3. EE

The total oil (TO) content was determined as previously described [15] with slight modifications. One gram of the powder was mixed with 5 mL of deionized water using a vortex mixer for 2 min and then extracted with 25 mL of hexane/propan-2-ol (3:1 *v*/*v*) for 5 min. The mixture was separated by centrifugation (Universal 320R; Hettich, Balingen, Germany) at 3000 rpm for 30 min. The clear upper part (organic phase) was collected and the lower aqueous phase was re-extracted using the same solvent mixture. The mixture was then filtered through a sodium sulfate column and the filtrate was evaporated under a fume hood. TO content was determined gravimetrically. For surface oil or free oil (FO), 15 mL of hexane was added to 2 g of the powder and mixed for 2 min. The mixture was then filtered through Whatman filter paper grade 1 and rinsed twice with 20 mL of hexane. The filtrate was then evaporated under a fume hood, and FO was determined by the mass difference. All measurements were performed in triplicate. EE was expressed as a percentage (%) and was determined using the following equation:

%EE = [(TO − FO)/TO] × 100(5)

#### 2.5.4. Morphology of Powder

The morphology of the spray-dried microcapsules was examined by scanning electron microscopy (SEM) using a JSM-IT300 microscope (JEOL, Tokyo, Japan). A small amount of powder was adhered to the stub using double-sided tape and coated with gold. The coated stub was examined under vacuum at a voltage of 10 kV and magnifications of 5000× *g*.

### 2.6. Stability Study of Encapsulated Powder

One formulation was selected from the experimental design for the stability study. The encapsulated powder was packed in an aluminum foil bag, sealed, and stored at 4 °C (control) or 40 °C/75% relative humidity (RH). Samples were randomly collected and analyzed for %EE and moisture content at designated time points of 0, 15, 30, 60, and 90 days. 

### 2.7. Statistical Analyses

All experiments were performed in triplicate and were statistically analyzed by analysis of variance (ANOVA), using Minitab software 19, followed by Tukey’s multiple comparisons test. A *p*-value < 0.05 was statistically significant.

## 3. Results and Discussion

### 3.1. Characterization of Emulsions

The results of all emulsion formulations are summarized in Table 1. All the emulsions remained stable for 24 h without phase separation. However, despite the droplet sizes, the emulsions prepared with a low wall-to-oil ratio of 1:1 (i.e., F1 and F3) presented oil droplets on the surface after 24 h. The emulsion droplet sizes ranged from 0.175 to 1.182 μm. The wall-to-oil ratio, type of wall material, and their interactions had a significant effect on the emulsion droplet size (*p* < 0.05). As the wall-to-oil ratio decreased, droplet size increased. This could be explained by the insufficient amount of wall material at a low wall-to-oil ratio (1:1). High oil-loaded emulsions were less viscous than low oil-loaded ones. This could affect the stability of the emulsion and result in larger droplet sizes [16]. Therefore, for F1 and F3 at the same low wall-to-oil ratio, F3 with the low viscosity of OSA starch/MD produced a larger droplet size (1.182 μm). However, no significant difference was observed between 2:1 and 3:1 for any type of wall material. Among the types of wall materials, WPI/MD produced the same emulsion droplet size regardless of the wall-to-oil ratio.

**Figure 1:**
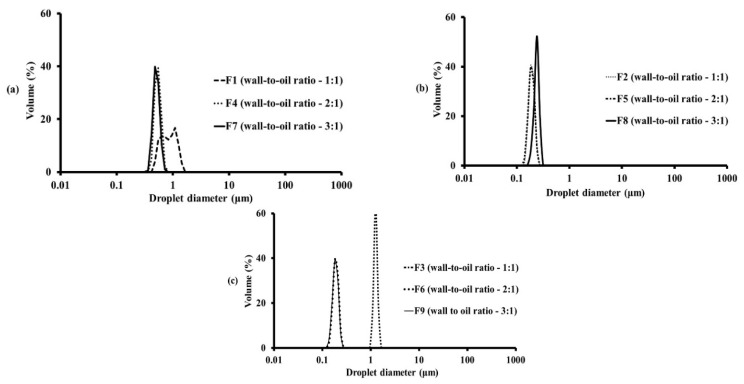
Droplet size and size distribution of emulsions prepared using different wall-to-oil ratios and types of wall materials: (**a**) GA/MD, (**b**) WPI/MD, and (**c**) OSA starch/MD with 40% total solid content.

The span values ranged from 0.186 to 0.807. A small span value indicates a homogeneously mixed emulsion. All the droplet sizes in this study showed monomodal distributions, except for F1 (bimodal distribution), as shown in Figure 1a. This might be due to the high oil loading and low amount of emulsifier in this formulation.

The viscosity results ranged from 11.75 to 96.36 mPa·s. The wall-to-oil ratio, type of wall material, and their interaction had significant effects on the emulsion viscosity (all *p* < 0.05). The viscosity of all emulsions gradually increased with increasing wall-to-oil ratios. However, the formulations with GA/MD showed very high viscosity when compared with WPI/MD or OSA starch/MD. These results agreed with previous findings that formulations using a combination of MD and GA showed higher emulsion viscosity when compared with the combination of MD and other polymers (i.e., Hi-Cap^®^, Capsul^®^, whey protein concentrate) [17,18,19]. 

The zeta-potential values ranged from |−30.50| to |−54.40| mV. WPI/MD displayed the highest zeta-potential value, followed by GA/MD and OSA starch/MD. The parameters that statistically significantly affected the emulsion zeta potential from greater to lesser effect were the type of wall material, the interaction of wall-to-oil ratio and type of wall material, and the wall-to-oil ratio (*p* < 0.05). In general, zeta potentials > |±30| mV can prevent emulsion droplets from aggregating and retain good stability [13,20]. The greater the zeta-potential absolute value, the higher the emulsion stability. All formulations in this study had a zeta-potential absolute value >30 mV, indicating good emulsion stability. It was difficult to explain the impact of the wall-to-oil ratio on the zeta potential, as all the results obtained by varying the wall-to-oil ratios were approximately the same for all types of wall materials. 

### 3.2. Characterization of Spray-Dried Powder

The yield ranged from 71.99% to 88.57%, as shown in Table 2. The yield was affected by the wall-to-oil ratio, type of wall material, and the interaction of these two factors (*p* < 0.05). The wall-to-oil ratio had a negative influence on the yield; as the wall-to-oil ratio increased, the yield decreased. Especially for GA/MD formulations, the higher yield of 85.07% (F1) was significantly decreased to 71.99% (F7) when the wall-to-oil ratio was increased from 1:1 to 3:1. Among all the formulations, the highest yield (88.57%) was achieved in F3 where the microcapsules were prepared using OSA starch/MD. F7, in which GA/MD was the wall material, produced the lowest yield (71.99%). In this scenario, the low viscosity of F3 (11.75 mPa·s) could be an asset for processing a higher yield than F7 (96.36 mPa·s), while both formulations were prepared with the same higher solid content (40%). This was similar to a previous report, as the high viscosity of the feed emulsion could prolong the drying time, resulting in a sticky powder that could affect the yield and increase loss [12]. 

The wall-to-oil ratio and the type of wall material had a significant effect on the moisture content (*p* < 0.05). As the wall-to-oil ratio increased, the moisture content of the microcapsules increased, but there was no statistically significant difference between the ratios of 2:1 and 3:1. Among the wall materials, the microcapsules prepared using OSA starch/MD showed a statistically higher moisture content than those prepared using WPI/MD and GA/MD. This may be due to the influence of the molecular size of the wall material, which may hinder the diffusion rate of particle crust formation [21]. A previous study demonstrated that there was no significant effect of different wall materials on the moisture content of coconut oil microcapsules, as all formulations contained the same amount of water [22]. The moisture content in a recent study ranged from 3.68% to 5.23%. This might be due to a higher solid content (>30%), which could increase the viscosity and retard water diffusion, resulting in microcapsules with high moisture content [23]. However, the moisture content of all the formulations was within 4–6%, which is considered appropriate for food powders [24].

The %EE results are shown in Table 2. The %EE ranged from 12.22% to 98.87%. The wall-to-oil ratio, type of wall material, and their interaction had a significant effect on %EE (*p* < 0.05). The %EE decreased with decreasing wall-to-oil ratio. This could be due to the insufficient amount of wall material to cover the formed droplets, which could increase the chance of droplet coalescence, especially with a low wall-to-oil ratio (1:1). This result agreed with previous findings that %EE was inversely affected by oil load [12,25,26]. F1 yielded the lowest %EE (12.22%). This formulation had no phase separation, but oil droplets were observed on the emulsion’s surface. This surface oil of initial emulsion might be one of the reasons for lowering the %EE. Among the types of wall materials, the highest %EE was found in F9 (98.87%), where MCT oil was encapsulated with OSA starch/MD at a high wall-to-oil ratio. However, no statistical difference was found between the wall-to-oil ratios of 2:1 and 3:1 in the OSA starch/MD formulations (F6 and F9). Similarly, there was no significant difference in %EE in the WPI/MD formulations with the low wall-to-oil ratios of 1:1 and 2:1 (i.e., F2 and F5). This could be due to the low viscosity of the OSA starch/MD and WPI/MD, which enabled the formation of small emulsion droplet sizes and prevented core leakage during spray drying, resulting in better protection. Generally, a wall-to-oil ratio between 2 and 4 (*w*/*w*) has been regarded as a suitable ratio, because a ratio below 2 could enhance surface oil formation, whereas a ratio above 4 could result in low loading capacity [27]. Since there was no statistically significant difference between the F9 (3:1) and F6 (2:1) formulations, F6 was selected for further analysis due to its higher oil-loading capacity.

**Figure 2:**
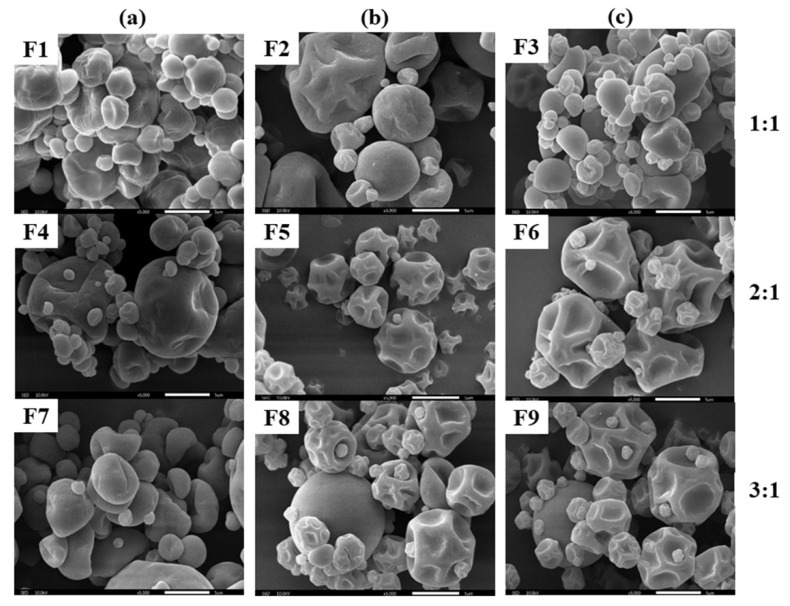
SEM images of microcapsules prepared with (**a**) GA/MD, (**b**) WPI/MD, and (**c**) OSA starch/MD at different wall-to-oil ratios at 40% total solid content. Magnification: 5000×.

**Figure 3:**
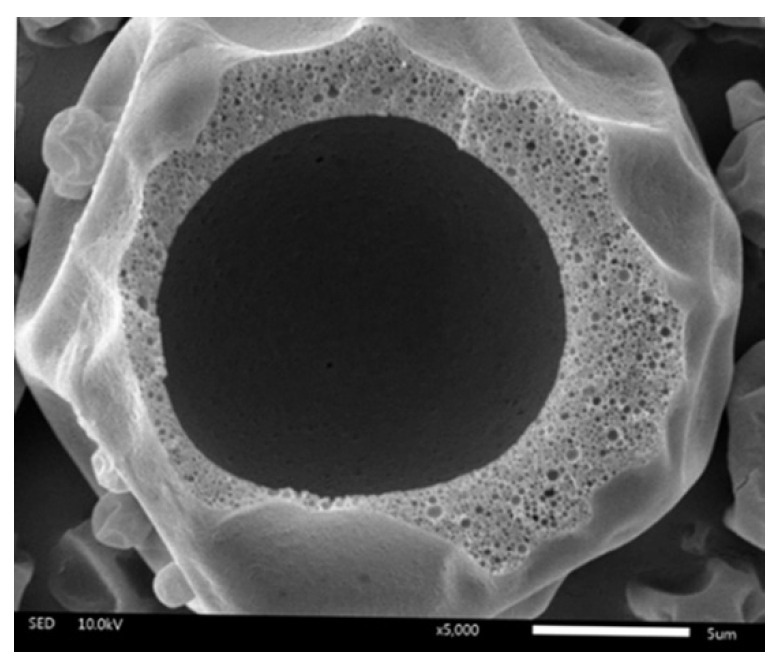
The inner structure of the microcapsule (F6) was prepared using OSA starch with a 2:1 wall-to-oil ratio and 40% total solid content. Magnification: 5000×.

Figure 2 displays representative SEM micrographs of all the spray-dried MCT microcapsules. Overall, the powders showed more or less dents with no surface cracks. The dents on the surface were correlated with prolonged film formation and the early stages of drying under low inlet temperature [17,28]. Differences in powder surface morphology were evident by comparing the extent of the shrinkage of the microcapsules. The shrinkage of the particle surface was obviously decreased when the wall-to-oil ratio was decreased from 3:1 (F7, F8, and F9) to 1:1 (F1, F2, and F3). This was due to highly loaded oil at 1:1, which leads to the formation of fatty skin on the droplet and prevents particle shrinkage [29]. Figure 3 presents the inner structure of the final selected formulation (F6). All MCT oil droplets were successfully embedded and evenly distributed inside the wall material and provided the best protection.

### 3.3. Stability Study of MCT Oil Encapsulated Powder

The formulation is intended for storage in a refrigerator due to the instability of MCT oil and WPI [30]. Therefore, the stability of the F6 formulation was investigated at 40 °C/75% RH for three months, compared to the control (4 °C). Table 3 presents the results. After storage for 15 days, the %EE was slightly decreased from 95.10% to 94.11% and 94.13% for the control and 40 °C/75% RH, respectively. After 90 days, the %EE was still approximately 94% under both storage conditions. The moisture content from the beginning to the end of the stability study was approximately 4–5%. These results indicate that F6 has good physical stability.

## 4. Conclusions

MCT microcapsules were successfully prepared by spray drying. SEM images showed the absence of surface cracks. The wall-to-oil ratio, type of wall material, and their interaction could affect the characteristics of the emulsion and microcapsule. An exception was the moisture content, where the interaction of wall-to-oil ratio and type of wall material was not found. A 1:1 wall-to-oil ratio for GA/MD or OSA starch/MD was not suitable for encapsulating MCT oil because some oil droplets were found on the surface of the emulsions. Concerning wall material, the OSA starch/MD formulations yielded a higher %EE than those of the WPI/MD and GA/MD formulations. The F6 formulation, in which OSA starch/MD was used as the wall material at a 2:1 wall-to-oil ratio, resulted in a small droplet size and a low-viscosity emulsion. The spray-dried powder had a high EE, acceptable moisture content, and stability during the stability study. In conclusion, OSA starch/MD is an appropriate wall material for encapsulating MCT oil by spray drying.

## Figures and Tables

**Table 1:** Characterization of emulsions.

	Factors		Responses				
Formula	X1	X2	Phase Separation	Droplet Size (μm)	Span	Viscosity (mPa·s)	Zeta Potential (mV)
F1	1	GA/MD	No *	0.831 ± 0.029 ^b^	0.807 ± 0.049	57.11 ± 0.75 ^c^	−43.47 ± 0.31 ^e^
F2	1	WPI/MD	No	0.196 ± 0.030 ^d^	0.268 ± 0.061	11.85 ± 0.08 ^g^	−50.17 ± 0.15 ^f^
F3	1	OSA starch/MD	No *	1.182 ± 0.003 ^a^	0.186 ± 0.004	11.75 ± 0.09 ^g^	−31.43 ± 0.68 ^b^
F4	2	GA/MD	No	0.465 ± 0.002 ^c^	0.546 ± 0.011	83.39 ± 0.52 ^b^	−42.43 ± 0.23 ^d^
F5	2	WPI/MD	No	0.200 ± 0.038 ^d^	0.384 ± 0.241	16.70 ± 0.06 ^f^	−53.60 ± 0.10 ^g^
F6	2	OSA starch/MD	No	0.177 ± 0.002 ^d^	0.374 ± 0.115	16.17 ± 0.22 ^f^	−30.50 ± 0.00 ^a^
F7	3	GA/MD	No	0.417 ± 0.050 ^c^	0.735 ± 0.354	96.36 ± 1.31 ^a^	−42.47 ± 0.35 ^d^
F8	3	WPI/MD	No	0.191 ± 0.025 ^d^	0.465 ± 0.202	20.81 ± 0.17 ^d^	−54.40 ± 0.40 ^g^
F9	3	OSA starch/MD	No	0.175 ± 0.001 ^d^	0.382 ± 0.105	19.07 ± 0.62 ^e^	−32.43 ± 0.06 ^c^

* After 24 h, oil droplets were observed on the emulsion’s surface. The phase separation of the emulsions was checked during 24 h. X1: wall-to-oil ratio, X2: type of wall material. Different superscript letters in the same column indicate significant differences (*p* < 0.05).

**Table 2:** Characterization of spray-dried powder.

Formula	Factors		Responses		
	X1	X2	Yield (%)	Moisture Content (%)	Encapsulation Efficiency (%)
F1	1	GA/MD	85.07 ± 0.10 ^ab^	3.68 ± 0.26	12.22 ± 1.52 ^e^
F2	1	WPI/MD	88.33 ± 0.59 ^ab^	3.75 ± 0.25	88.82 ± 0.10 ^c^
F3	1	OSA starch/MD	88.57 ± 0.46 ^a^	4.45 ± 0.41	83.92 ± 0.19 ^d^
F4	2	GA/MD	82.54 ± 3.52 ^b^	3.89 ± 0.16	83.95 ± 1.36 ^d^
F5	2	WPI/MD	86.51 ± 2.41 ^ab^	4.03 ± 0.28	89.60 ± 0.29 ^c^
F6	2	OSA starch/MD	84.83 ± 3.98 ^ab^	4.95 ± 0.10	98.38 ± 0.01 ^a^
F7	3	GA/MD	71.99 ± 0.77 ^c^	4.06 ± 0.06	90.69 ± 0.03 ^c^
F8	3	WPI/MD	82.60 ± 0.26 ^b^	4.37 ± 0.27	96.03 ± 0.03 ^b^
F9	3	OSA starch/MD	84.22 ± 1.52 ^ab^	5.23 ± 0.07	98.87 ± 0.19 ^a^

X1: wall-to-oil ratio, X2: type of wall material. Different superscript letters in the same column indicate significant differences (*p* < 0.05).

**Table 3:** Stability of microcapsule (F6) at control and 40 °C/75% relative humidity (RH) for three months.

Days	Encapsulation Efficiency (%)		Moisture Content (%)	
	Control	40 °C/75% RH	Control	40 °C/75% RH
0	95.10 ± 0.03	95.10 ± 0.03	4.95 ± 0.10	4.95 ± 0.10
15	94.11 ± 0.10	94.13 ± 0.30	5.23 ± 0.06	5.29 ± 0.13
30	94.24 ± 0.02	94.13 ± 0.02	4.62 ± 0.31	4.86 ± 0.32
60	94.23 ± 0.02	94.19 ± 0.06	4.65 ± 0.21	4.31 ± 0.34
90	94.14 ± 0.02	94.20 ± 0.04	4.35 ± 0.12	4.18 ± 0.34

## Data Availability

Not applicable.
